# Measuring the Prevalence of Adverse Childhood Experiences by Survey Research Methods

**DOI:** 10.3390/ijerph16061048

**Published:** 2019-03-22

**Authors:** Anikó Ujhelyi Nagy, Ildikó Kuritár Szabó, Endre Hann, Karolina Kósa

**Affiliations:** 1 Institute of Behavioral Sciences, Faculty of Public Health, University of Debrecen, Móricz Zsigmond krt. 22., H-4032 Debrecen, Hajdú-Bihar, Hungary; szabo.ildiko@sph.unideb.hu (I.K.S.); kosa.karolina@sph.unideb.hu (K.K.); 2 Median Opinion & Market Research Ltd., Fürj utca 2., H-1124 Budapest, Hungary; hann@median.hu

**Keywords:** adverse childhood experiences (ACEs), Hungarian representative adult sample, opinion poll, ACE Score Calculator

## Abstract

*Background*: Child maltreatment has been firmly established as a fundamental risk factor for adult health. However, its quantification poses many questions methodologically, psychologically, and culturally alike. We carried out the first nationally representative survey research in Hungary and in Central–Eastern Europe to assess the prevalence of adverse childhood experiences (ACEs) among adults. *Methods*: Data were collected by an opinion research company using a screening tool of the Adverse Childhood Experiences study. *Results*: 25% (n = 293) of adults reported any childhood adversity; 5% (n = 59) of them had four or more ACEs. The most prevalent forms of child maltreatment were emotional (5%, n = 59) and physical abuse (5%, n = 59), sexual abuse (1%, n = 12) being the least prevalent. The most frequent dysfunctional household condition was parental divorce or separation (13%, n = 153), followed by household substance abuse (11%, n = 129). *Conclusions*: Nationally representative surveys on ACEs found a range of overall prevalence of various forms of child maltreatment between 14.1 and 35.2% into which our results fall. Nevertheless, our survey most likely underestimates the prevalence of child maltreatment in Hungary, reflecting the impact of a host of factors influencing awareness. Survey research methods are appropriate to obtain nationally representative data on child maltreatment that not only contribute to designing interventions but can also be used to monitor the effectiveness of interventions to improve child and adult health in the long run.

## 1. Introduction

Exposure to various forms of adversity early in life has been shown to lead to an increased risk of a broad range of developmental difficulties, principally cognitive, emotional, and behavioral impairments during childhood that are mediated by compromised neurodevelopment affecting various parts of the brain [1,2,3,4,5,6,7]. The consequences of childhood maltreatment can last well into adulthood or even throughout life, impacting adult physical health, mental pathology, and quality of life [8,9,10,11]. Numerous studies have shown that adverse childhood experiences (ACEs, including forms of child maltreatment and household dysfunctions) are major risk factors for acute and chronic somatic and mental diseases such as anxiety or post-traumatic disorders mediated by risk behaviors such as smoking, alcohol and drug abuse, suicide attempts, aggressive behaviors, risky sexual behaviors, and low mental resilience [12,13,14,15,16,17,18,19,20]. Previous studies provided strong evidence that ACEs tend to co-occur in which intergenerational transmission of adversity might be a contributing factor [21,22,23,24].

Prevention of these early adversities is much more effective than treatment of their consequences with their enormous burden in health and social care, as well as in the education system [25,26]. National policies and evidence-based prevention programs (at local and societal levels) based on early recognition of ACEs may contribute to preventing a wide range of health-harming behaviors, somatic and mental disorders, and early death [15,27,28]. All such policies, programs, and interventions should be based on an in-depth knowledge of the population pattern of ACEs. However, collecting relevant information has been hindered either by lack of awareness about the issue and/or by a lack of relatively simple and cost-effective methods of collecting information in various population groups.

### Tested Methodologies for Studying Childhood Adversity

The causal relationship between childhood adversity and its adult health consequences, including mental and somatic health impairments, have been established by prospective longitudinal cohort studies such as the Lehigh Longitudinal Study of the US established in 1976 [29], the Christchurch Health and Development Study established in 1977 [30], and the Adverse Childhood Experiences (ACE) Study in 1995 [15]. The majority of research collected information on childhood adversity either from the primary caretaker of the child in cases of prospective studies or from adult self-reports in terms of their childhood in retrospective or cross-sectional studies. Retrospective assessment of ACEs based on self-report was shown to be reliable and valid for research purposes [31,32,33]. Retrospective recall of ACEs can be considered valid if these experiences are operationalized unequivocally, making interpretation and judgment of the questions unnecessary [31,32]. Data can be collected in various ways such as by questionnaire during personal interview [30,34,35]; mailing the questionnaire to respondents by post or by email [15]; or by telephone interviews [36,37].

**Figure 1:**
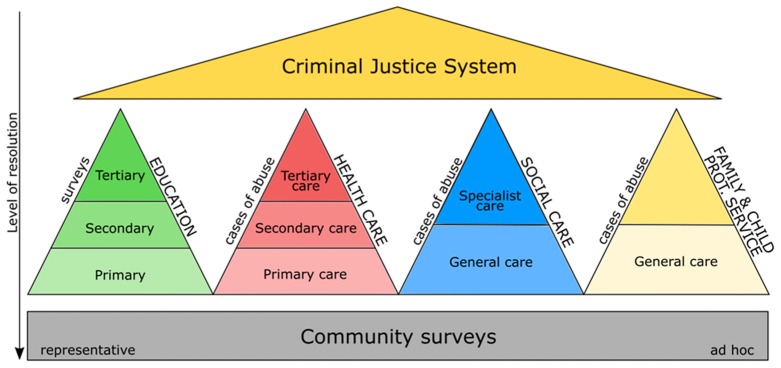
Sources of data for child maltreatment.

In order to make an evidence-based statement about the pattern of childhood adversity in any given population, survey research should be designed producing reliable population estimates from samples that represent the entire population of interest. A practical handbook on measuring and monitoring national prevalence of child maltreatment published by the World Health Organization promotes system-wide monitoring of child maltreatment in European countries and globally with the emphasis on estimating population-wide prevalence rates based on representative survey samples [38]. However, many studies reporting child maltreatment rely on clinical and other nonrepresentative samples drawn from various public services such as education, health care, social services, or family and child protective services that make the generalization of findings difficult (Figure 1) [39].

So far, only one research study has been published in the literature that assessed childhood adversity in a nationally representative sample in which fieldwork was carried out by a government-financed agency. Namely, the second wave (2004–2005) of the National Epidemiological Survey on Alcohol and Related Conditions (NESARC) in the US collected, among others, data on adverse childhood events by face-to-face interviews conducted by trained lay interviewers of the US Census Bureau in a nationally representative adult sample of 34,653 persons from the United States. Based on these data, the prevalence of emotional abuse (4.8%) and of emotional neglect (6.2%) were estimated [35].

The ACE study was initiated by a health insurance organization among a subset of its clients, and its ongoing surveillance is limited to the participants of the original study [40].

The Behavioral Risk Factor Surveillance System (BRFSS) of the Centers for Disease Control and Prevention (CDC) in the US has been collecting data about adverse childhood experiences since 2009 by the request of individual states of which 32 requested such data collection [41].

Survey research, that is, data collection from a carefully selected nationally representative sample, requires human and financial resources that are beyond the reach of academic institutes, or even governmental agencies in most countries—save for the US. On the other hand, polling companies have vast survey research experience gathering information on a wide range of topics. This experience was taken advantage of in two European studies that used survey research methods to study the epidemiology of ACEs in nationally representative samples. One of the studies was carried out on a representative sample of 2504 German participants between 14 and 92 years by face-to-face interviews on childhood abuse and neglect, as well as current anxiety and depression. Data collection was carried out by an independent institute for opinion and social research [42]. The other study was done in a sample of 3885 adults representative of England in which information on childhood experiences and adult mental well-being was collected during personal visits by a professional survey company directed by researchers [43].

Encouraged by these antecedents, our aim was to obtain data on the prevalence of adverse childhood experiences in the adult Hungarian population in line with the recommendations of the World Health Organization using opinion research methodology.

## 2. Materials and Methods

### 2.1. Sampling and Data Collection

A market research company (Median Opinion and Market Research Institute) was contracted based on its outstanding performance predicting election results in Hungary. A multistage stratified cluster sampling was carried out using the most recent census list (2011) of the Central Statistical Office of Hungary. Based on detailed maps of the country, 120 sampling units were selected by a computer program of Median Opinion and Market Research Institute. Sampling units represented the entire territory of the country according to EUROSTAT NUTS II levels and according to the distribution of the resident population in terms of metropolitan, urban, and rural areas. One starting address was randomly drawn in each selected sampling unit from which nine other households were accessed by random walking (10 households per sampling unit). One respondent 18 years or older was interviewed in each household by using the Kish selection grid [44]. Each selected person was contacted in person at least three times if the first attempt was not successful. In case of refusal, the interviewer had to select another respondent in another household based on a preset algorithm.

General questions were asked face to face, whereas the questions relating to childhood adversity were filled by the respondents themselves. The interviewer handed over the paper-based questionnaire to the respondent in person. The respondents were allowed to submit their responses in a sealed envelope upon request; 10 % of the completed interviews were validated by face-to-face or telephone re-interview.

Altogether, 1200 persons aged 18 years or older were interviewed out of 1608 who were attempted to be interviewed (74.6% response rate). All interviewees received a written statement about data collection being voluntary and conforming to the requirements of the national data protection act; none of them received incentive in any form. Data collection was carried out by trained interviewers in person in March 2016.

Median Opinion and Market Research Institute is one of leading research companies in Hungary, conducting high-quality market, opinion, and social research. The institute follows the professional and ethical guidelines specified in the ESOMAR Code of Conduct [45]. During the present research, informed consent was provided and the appropriate ethical standards (according to the World Medical Association Declaration of Helsinki) were followed. The protocol of research was approved by the Medical Research Council of the University of Debrecen (4499-2015).

### 2.2. Measures

#### 2.2.1. Sociodemographic Variables

Age, gender, marital status (unmarried, married, registered partnership, divorced, widow), type of the settlement of permanent residence (capital, city, village), education (less than primary, primary, vocational, high school diploma, college/university), type of work (manual vs nonmanual), employment (nine categories), and current household income (four quartiles) were registered. Sociodemographic categories were identical to those used by the Central Statistical Office of Hungary.

#### 2.2.2. Adverse Childhood Experiences

Adverse childhood experiences were assessed by the ACE Score Calculator, a validated screening instrument used to estimate the prevalence of ACEs [46]. This tool of 10 items, developed by the researchers of the ACE study based on the original ACE Questionnaire, is appropriate for screening purposes and allows individuals to calculate their own scores based on the original scoring criteria of the ACE Study [15]. A short form of eight items of the original ACE Questionnaire was also used in the Health Behavior in School-Aged Children (HBSC) Study and proved to be reliable [38].

The ACE Score Calculator helps assess exposure to 10 types of ACEs including 5 types of abuse (emotional, physical, and sexual), neglect (physical and emotional), and 5 types of dysfunctional family environment (mentally ill or substance-abusing member of household, physical violence in the household, parental separation/divorce, incarcerated family member(s) prior to age 18). The ACE Score is calculated by summing up all 10 ACE variables and serves as a measure of overall ACE exposure ranging from 0 (meaning no exposure to the 10 categories of ACEs) to 10 (meaning exposure to all 10 categories). Responses were categorized by type of ACE and were dichotomized into no history or any history of adversity prior to age 18. Responses were analyzed also by number of adverse experiences (none, 1, 2–3, 4 or more) prior to age 18.

Dube et al. (2014) found good to excellent reliability in the reports of ACEs during adulthood. The test–retest reliability in the responses to questions about ACEs and the resulting ACE score was found to be good and moderate to substantial. These findings confirm that retrospective responses to the forms of childhood maltreatment and household dysfunction are generally stable over time [31]. Wingenfeld et al. (2010) investigated the psychometric characteristics of the ACE Score Calculator and revealed that it is a reliable, valid, and economic screen for the retrospective assessment of ACEs [47].

The English version of the ACE Score Calculator was translated to Hungarian by the authors, and cross-cultural adaptation was carried out through an iterative forward–backward translation compared by an independent third person. The preambles, item contents, and response options for items can be found in the Appendix A (Table A1).

### 2.3. Data Analysis

In order to obtain estimates of adverse childhood experiences in the adult noninstitutionalized population of Hungary, statistical weights were applied to ensure that estimates reflect the general adult Hungarian population gender, age group, education, settlement type, and region. The sample defined as survey sample was analyzed using Stata/IC 13.1. Single-stage design was used stratifying the sample based on the sampling units, that is, regions of the country. The Taylor method was used to estimate sampling errors; primary sampling units were sampled without replacement [48]. Analysis of variance was computed to examine the prevalence of child maltreatment and household dysfunctions by total ACE core and by type of ACE stratified by gender. The sociodemographic characteristics of respondents reporting no history (ACE score = 0) or any history of adversity (ACE score >0) were described defining the ACE score as a discrete interval variable. The number of categories of demographic variables such as education and employment type was combined to reduce the number of categories and to simplify interpretation. Considering the weighted estimates, all prevalence data were rounded.

Logistic regression was carried out by backward stepwise regression to identify the independent variables of childhood adversity. The ACE score was defined as the binary outcome variable as described above (no childhood adversity vs any history of adversity). One binary ACE score was also created considering only the five types of childhood maltreatment, and another by including only the five types of family dysfunction. Age (in years), sex (female, male), place of residence (capital, city, village), education (higher education vs less), type of work (nonmanual vs manual), and marital status (single, divorced, married, cohabiting, widowed) were tested as categorical explanatory variables. In terms of marital status, two models were tested. The first model compared those in an ongoing relationship (married or cohabiting) to all other marital categories (single, divorced, widowed) including all respondents. The second model compared those in an ongoing relationship (married or cohabiting) to those who ended their relationship (divorced) including only those with a (supposedly) living present or past partner. Post-test analysis was carried out by the adjusted Wald test.

## 3. Results

### 3.1. Sample

Altogether, 1200 persons aged 18 years or older were interviewed, representing 0.012% of the Hungarian adult noninstitutionalized population according to the census in 2011. Respondents ranged in age from 18 years to 112 years and the mean age for the sample was 53.2 (SD = 16.5) years; 37.65% of the respondents were men. The sample was weighted to represent the Hungarian adult population by gender, age group, education, settlement type, and region. Of all the persons who had been approached, 74.6% were willing to fill out the questionnaire. Twenty-six individuals filled out the screening instrument incompletely: 17 did not complete the full questionnaire, 9 respondents answered all but one question. They were dropped from the analysis, leaving a total sample size of 1174 corresponding to a completion rate of 97.8%.

### 3.2. The prevalence of Adverse Childhood Experiences in Hungary

The distribution of sociodemographic characteristics overall and by reporting an ACE (no ACE vs at least one ACE) is provided in Table 1. The highest prevalence of any adversity, 28% (n = 82) was found in the youngest age group (18–29 years) that was declining and was half of that among the 50–59-year-olds, but somewhat increased in the oldest age group (60+, 23%, n = 67). Experience of childhood adversity was more than twice as high in cities compared to the capital or to villages. Interestingly, the ACE score was by far the lowest among the least-educated group and highest among those with high school qualification. Income was mildly significantly related to the experience of adversity: those in the lowest income quartile had the highest proportion of any adversity. One-quarter more of those who suffered any ACE had been unmarried or divorced compared to persons who did not report any ACE.

Of the adult Hungarian population, 25% (n = 293) reported having experienced some kind of childhood adversity before the age of 18 years; 5% (n = 59) of the respondents had four or more ACEs. There were no significant gender differences regarding the co-occurrence of ACEs (Pearson’s chi-squared test, p = 0.29) (Table 2). Considering only those between the ages of 18 and 80 years, the prevalence of any abuse did not change (25%).

The most prevalent form of self-reported child maltreatment was emotional abuse (5%, n = 59), and physical abuse (5%, n = 59) in this nationally representative sample. The least prevalent pattern was sexual abuse (1%, n = 12). The most frequent dysfunctional household condition was parental divorce or separation (13%, n = 153), followed by household substance abuse (11%, n = 129). The least prevalent household dysfunction was having an incarcerated household member (4%, n = 47).

Among women, emotional abuse and physical abuse were more prevalent (7% (n = 51) for emotional abuse and 6% (n = 44) for physical abuse) than among men (4% (n = 18) for physical abuse and 3% (n = 13) for emotional abuse). Male participants witnessed household physical violence more often (violence against their mother or stepmother) according to their self-report. Gender differences in emotional abuse have been shown to be significant (Pearson’s chi-squared test, *p* = 0.03) (Table 3).

Respondents having experienced four or more ACEs were younger (57% (n = 29) were 18–39 years old), more of them lived outside the capital (75% (n = 38) lived in cities or villages), belonged to the lowest income category (42%, n = 21), were married (49%, n = 24), and had no children (43%, n = 21) (Table 4).

### 3.3. Modeling the Determinants of Adverse Childhood Experiences in Hungary

Independent determinants of adverse childhood experiences were analyzed by logistic regression defining childhood abuse as a binary variable (experienced vs not experienced). Any childhood maltreatment (5 types of abuse or neglect of any sort), family dysfunction (5 types), and both combined, that is, any adverse experience (10 types), as described in Methods, as outcome variables were modeled. Marital status was defined in three different ways. In Model 1, all respondents were divided into two categories: those in an actual relationship (married or cohabiting) and those not currently in a relationship (single, divorced, widowed) (Table 5). One quartile increase of income decreased the odds of reporting any childhood maltreatment by 24% in Model 1, but income did not remain a significant determinant of reporting childhood adversity in either of the models.

In Model 2, only persons with a living present or past partner were included. Those in actual relationships (married or cohabiting) were compared to those who were divorced (Table 6). The latter produced a better model in which living in a relationship decreased the odds of reporting maltreatment by 35%. In the same model, one year increase in age decreased the odds of reporting any childhood maltreatment by 2.3%, and living in a city compared to a village increased the odds of reporting any maltreatment by 76%. Age, type of permanent residence, and marital status were found to be independent determinants of family dysfunction or any adverse childhood experience in the best adjusted logistic regression model (Model 2).

In Model 3, persons with relationship experience were included. Those in actual relationships (married or cohabiting) were compared to those who were divorced or widowed (Table 6). Model 2 and 3 both were statistically significant, showing that currently living in a relationship decreased the odds of reporting any childhood adversity by at least 40% compared to those who had relationship experience but did not currently live in one.

Other variables such as education, gender, number of children, type of work (manual, nonmanual) were not found to be significant determinants of reporting any childhood maltreatment, any family dysfunction, or a combination of both (data not shown).

## 4. Discussion

Our research produced the first national and the third European representative survey on adverse childhood experiences in Hungary according to which 25% (n = 293) of the Hungarian general population reported experiencing any childhood adversity before the age of 18 years with no gender difference; 5% (n = 59) of the respondents had four or more ACEs. The most prevalent form of child maltreatment was emotional (5%) and physical abuse (5%, n = 59); sexual abuse (1%, n = 12) was least prevalent. Parental divorce or separation (13%, n = 153), followed by household substance abuse (11%, n = 129) were the most frequent dysfunctional household conditions. The higher prevalence of ACEs among the youngest age group of adults may indicate an increasing awareness due to a more open public attitude and changing public opinion.

Our study is the first survey on adverse childhood experiences in a nationally representative adult sample of the Hungarian population; the first survey in any Central–Eastern European country; and the third such survey in developed countries that used a marketing research company for data collection. The European Commission has already established the feasibility of using marketing research/opinion polling agencies in health research: two reports were published on various aspects of the mental health of the population of EU member states in 2006 and 2010 by contracting companies to carry out representative surveys with multistage probability sampling and face-to-face interviews [49,50].

In order to interpret our results, data from the other two representative national surveys cited in the Introduction were considered. As it is shown in Table 7, the occurrence of the most frequent ACEs substantially varies in these countries, with Hungary having the lowest and Germany the highest prevalence.

The NESARC and BRFSS surveys have unique features (study design and implementation by public agencies funded by the federal government of the US) based on phone interviews, repeated measures that may not be easily copied by other countries.

The strengths of our study include the use of an international standardized screening tool (ACE Score Calculator) in a nationally representative adult sample. Sampling and data collection were carried out by an experienced opinion poll company that used refined and tested sampling methods and had trained interviewers with experience in face-to-face data collection. This not only increased the reliability of data but was also cost-effective.

However, the study has limitations as well. The cross-sectional design and the retrospective nature of data collection limits the scope of interpretation; low awareness of the topic in the country probably increases recall bias, especially among older persons. However, since the prevalence of childhood maltreatment did not change significantly when those above the age of 80 years were removed from the analysis, and since the items of the ACE questionnaire are quite specific, not requiring interpretation, recall bias likely did not influence our results. The interference of dissociative defense mechanisms with recall cannot be excluded, but this bias cannot be avoided by any questionnaires. The conspicuously low frequency of childhood adversity among those with the lowest education merits further investigation.

In order to further probe the comparability of our data, the literature was searched for meta-analyses on the prevalence of child maltreatment and dysfunctional households reported by adults (Table 8). According to Stoltenborgh et al., global estimates of the prevalence in self-report studies were 22.6% for physical abuse, 36.3% for emotional abuse, 12.7% for sexual abuse (7.6% among boys and 18.0% among girls), 16.3% for physical neglect, and 18.4% for emotional neglect. These authors opined that the prevalence of child maltreatment seems to be largely similar across the globe. However, this statement is based mostly on research in western countries, mainly in North America and Europe [52].

Some meta-analyses were identified which focused on the prevalence of child maltreatment and dysfunctional households reported by children (Table 9).

The WHO Regional Office for Europe used 105 prevalence estimates from 50 community surveys to estimate the prevalence of sexual abuse as 9.6% (13.4% in girls and 5.7% in boys), physical abuse 22.9%, and emotional abuse 29.1% with no gender difference in the two latter types of abuse. The few studies that focused on neglect found high prevalence: 16.3% for physical and 18.4% for emotional neglect. As Table 8 shows, there are no differences between global and European prevalence estimates considering the majority of forms of maltreatment—the only exception being female sexual abuse with slightly lower prevalence in Europe [53]. The European report opined that prevalence estimates of child maltreatment would be higher in Eastern Europe. However, Gilbert et al. (2009) reported prevalences with a much greater variability in high-income countries: 3.7–16.3% of children experienced parental violence per year, 10.3% suffered from emotional abuse, and 1.4–15.7% suffered from neglect [27].

Our population survey measured a considerably lower prevalence of childhood adversity compared to population surveys in Germany or England. This, on one hand, probably reflects underestimation, supported by other data such as the homicide rate under 15 years of age in Hungary that was as high as 0.89 per 100,000 children or the fact that Hungary ranked 23rd out of 27 developed countries based on deaths due to abuse and/or neglect per 100,000 children under the age of 15 [53,59,60]; or that satisfaction with life among young teenagers was the second lowest in Hungary out of 21 developed countries in 2013 [61].

On the other hand, the widely different methods and measurements in various samples (community, clinical, and chance samples) selected by a wide variety of methods severely restricts the comparability of surveys carried out in different countries.

Third, the strong influence of culture, traditions, and religion on the treatment of children including what counts and what does not as maltreatment [62], as well as the possibility of false-negative statements due to psychological motives, must also be taken into account when comparing data on child maltreatment in various countries [32]. The ACE study was seminal in drawing attention to childhood adversity in the US and other developed countries [63], but this topic only recently has commanded attention in Hungary, reflected by the fact that no community-based data collection on childhood adversity had been carried out in the country.

Taking all these points together, the statement of Stoltenborgh and his coauthors (2015) that the prevalence of child maltreatment seems to be largely similar across the globe must be called into question [52]. Moreover, the opposite seems to be likely, which points to the importance of population- or community-based prevalence estimates measured by consistent methodology in each country.

## 5. Conclusions

Our survey provides a population-based set of reference data upon which a strategy to address childhood adversity should be built and to which future data can be compared. Considering the fact that 1) the design and implementation of national surveys is beyond the resources of Hungarian academic institutes, 2) to our knowledge, no similar survey is being designed or planned by national institutions of public health or child protection, and 3) clinical samples have been known to overestimate the population prevalence of ACEs [39], marketing research methods provide a viable and cost-effective alternative to collect data on this important topic.

Even underestimated population-based data on childhood adversity are better than estimates based on clinical or chance samples or no data at all. Our survey provides the first data on ACEs in Central and Eastern Europe with the aim of advocating for the monitoring of ACEs in the future for which the use of marketing research methods seems to be appropriate. The European report on preventing child maltreatment states that community surveys using international standardized tools should be conducted regularly in order to identify the changes in prevalence rates and the potential risks and to have the opportunity to evaluate the implemented prevention programs [53]. However, until international standardized methods of measuring childhood adversity are developed, countries should aim at quantifying this important public health problem in a scientifically acceptable way for which less or more complex methods are available [64,65,66], and keep monitoring its tendency in time. If there is an issue in which national surveillance is more important than international comparability, it is childhood adversity, especially considering its long-term impact on the population’s well-being.

## Figures and Tables

**Table 1:** Distribution of sociodemographic characteristics by reporting/not reporting an ACE.

Sociodemographic Variable	Did not Report an ACE (*N* = 904) *N* (%)	Reported an ACE (*N* = 292) *N* (%)		Total (*N* = 1196) *N* (%)		*p*-Value ^a^
Age group						
18–29	127 (14)	82 (28)		209 (17)		0.0003 *
30–39	163 (18)	55 (19)		218 (18)		
40–49	154 (17)	50 (17)		204 (17)		
50–59	181 (20)	38 (13)		219 (18)		
60+	280 (31)	67 (23)		347 (30)		
Type of the settlement of permanent residence						
Capital	154 (17)	58 (20)		212 (18)		0.04 *
City	470 (52)	162 (55)		632 (53)		
Village	280 (31)	72 (25)		352 (29)		
Education						
Less than 8 grades	18 (2)	9 (3)		27 (2)		0.265
Primary school	181 (20)	44 (15)		225 (19)		
Vocational training	262 (29)	96 (33)		358 (30)		
High school	271 (30)	105 (36)		376 (31)		
College graduate	172 (19)	38 (13)		210 (18)		
Type of work						
Manual	515 (57)	181 (62)		696 (58)		0.647
Nonmanual	389 (43)	111 (38)		500 (42)		
Income quartiles						
Low	199 (22)	85 (29)		284 (24)		0.035 *
Lower middle	244 (27)	70 (24)		314 (26)		
Upper middle	181 (20)	67 (23)		248 (21)		
Upper	280 (31)	70 (24)		350 (29)		
Number of children						
0	660 (73)	184 (63)		844 (71)		0.161
1	118 (13)	55 (19)		173 (14)		
2	81 (9)	38 (13)		119 (10)		
3	36 (4)	12 (4)		48 (4)		
>4	9 (1)	3 (1)		12 (1)		

^a^ Indicates the application of the Pearson’s chi-squared test, * *p* < 0.05.

**Table 2:** Distribution of ACE Score overall and by gender.

ACE Score	Women (N = 732) N (%)	Men (N = 442) N (%)	Total (N = 1174) N (%)	*p*-Value ^a^
0	542 (74)	336 (76)	881 (75)	*p* = 0.29
1	88 (12)	53 (12)	141 (12)	
2	44 (6)	18 (4)	59 (5)	
3	22 (3)	13 (3)	34 (3)	
≥4	36 (5)	22 (5)	59 (5)	

^a^ Indicates the application of the Pearson’s chi-squared test, *p* = 0.29.

**Table 3:** Reported prevalence of ACEs in the sample and by gender.

Adverse Childhood Experiences (ACEs)	Women (N = 732) N (%)	Men (N = 442) N (%)	Total (N = 1174) N (%)	*p*-Value ^a^
Maltreatment				
Emotional abuse	51 (7)	13 (3)	59 (5)	0.03 *
Physical abuse	44 (6)	18 (4)	59 (5)	0.09
Sexual abuse	15 (2)	0 (0)	12 (1)	
Emotional neglect	29 (4)	18 (4)	47 (4)	0.54
Physical neglect	22 (3)	9 (2)	35 (3)	0.68
Family dysfunction				
Parental separation/ divorce	102 (14)	53 (12)	153 (13)	0.45
Household physical violence	29 (4)	27 (6)	59 (5)	0.12
Household substance abuse	95 (13)	44 (10)	129 (11)	0.16
Household mental illness	44 (6)	18 (4)	59 (5)	0.27
Incarcerated household member	29 (4)	18 (4)	47 (4)	0.69

^a^ Indicates the application of the Pearson’s chi-squared test, * *p* < 0.05.

**Table 4:** Distribution of sociodemographic characteristics by ACE Score.

Sociodemographic Variable	ACE Score = 0 (N = 904) N (%)	ACE Score = 1 (N = 153) N (%)	ACE Score = 2–3 (N = 89) N (%)	ACE Score ≥ 4 (N = 50) N (%)
Age group				
18–29	127 (14)	44 (29)	20 (23)	14 (27)
30–39	163 (18)	20 (13)	20 (23)	15 (30)
40–49	154 (17)	29 (18)	15 (16)	6 (13)
50–59	181 (20)	20 (13)	13 (15)	6 (13)
60+	280 (31)	40 (26)	21 (24)	9 (17)
Type of the settlement of permanent residence				
Capital	154 (17)	35 (23)	11 (12)	12 (25)
City	470 (52)	83 (53)	60 (67)	19 (38)
Village	280 (31)	35 (23)	18 (21)	19 (37)
Education				
Less than 8 grades	18 (2)	2 (1)	3 (4)	2 (5)
Primary school	181 (20)	23 (15)	15 (16)	6 (12)
Vocational training	262 (29)	45 (30)	31 (35)	18 (36)
High school	271 (30)	63 (41)	30 (34)	14 (28)
College graduate	172 (19)	20 (13)	10 (11)	10 (19)
Type of work				
Manual	515 (57)	86 (56)	65 (73)	30 (59)
Nonmanual	389 (43)	67 (44)	24 (27)	20 (41)
Income quartiles				
Low	199 (22)	35 (23)	25 (28)	21 (42)
Lower middle	244 (27)	35 (23)	22 (25)	2 (23)
Upper middle	181 (20)	40 (26)	24 (27)	5 (11)
Upper	280 (31)	43 (28)	18 (20)	12 (24)
Marital status				
Married	389 (43)	43 (28)	31 (35)	24 (49)
Registered partnership	63 (7)	9 (6)	10 (11)	8 (17)
Divorced	145 (16)	25 (23)	18 (21)	5 (11)
Widow	118 (13)	14 (21)	10 (9)	7 (4)
Unmarried	190 (21)	29 (45)	23 (20)	17 (9)
Number of children				
0	660 (73)	112 (73)	54 (61)	21 (43)
1	118 (13)	21 (14)	26 (29)	10 (19)
2	81 (9)	15 (10)	7 (8)	11 (22)
3	36 (4)	5 (3)	2 (2)	5 (11)
>4	9 (1)	0 (0)	0 (0)	3 (5)

**Table 5:** Independent determinants of experiencing any of the five types of maltreatment or five types of dysfunctions in the family (Model 1).

Sociodemographic Variables	Any Childhood Maltreatment			Family Dysfunction			Maltreatment & Family Dysfunction Together		
	OR	*p*-Value	Model	OR	*p*-Value	Model	OR	*p*-Value	Model
**Model 1: All respondents**									
Age (year)	1.000	0.925	F = 2.19 *p* = 0.068	0.979	0.000	F = 7.53 *p* < 0.001	0.983	0.002	F = 5.99 *p* < 0.001
City (ref: village)	1.376	0.226		1.765	0.007		1.546	0.026	
Living in relationship -married or cohabiting (ref: not in relationship)	0.827	0.409		0.726	0.079		0.698	0.038	
Income (quartiles)	0.759	0.015		0.874	0.092		0.894	0.141	

**Table 6:** Independent determinants of experiencing any of the five types of maltreatment or five types of dysfunctions in the family (Model 2 and Model 3).

	Any Childhood Maltreatment			Family Dysfunction			Maltreatment & Family Dysfunction		
Sociodemographic Variables	OR	*p*-Value	Model	OR	*p*-Value	Model	OR	*p*-Value	Model
**Model 2: Only respondents in current relationship or divorced**									
Age (year)	0.981	0.104	F = 1.88 *p* = 0.132	0.968	0.000	F = 8.55 *p* < 0.001	0.973	0.001	F = 7.32 *p* < 0.001
City (ref: village)	1.464	0.259		2.032	0.006		1.761	0.020	
Living in relationship-married or cohabiting (ref: divorced)	0.652	0.139		0.607	0.025		0.571	0.009	
**Model 3: Only respondents in current relationship or divorced/widowed**									
Age (year)	0.984	0.103	F = 1.73 *p* = 0.158	0.97	0.000	F = 10.03 *p* < 0.001	0.974	0.000	F = 8.13 *p* < 0.001
City (ref: village)	1.469	0.193		1.981	0.002		1.706	0.011	
Living in relationship-married or cohabiting (ref: divorced)	0.701	0.17		0.58	0.006		0.565	0.003	

**Table 7:** Prevalence of some ACEs in representative national samples.

	Germany	England	Hungary
Year of survey	2010	2013	2016
Tool	CTQ (28 items)	ACE screening tool (11 items)	ACE screening tool (10 items)
Age group	14–92	18–69	18–112
Sample size	2504	3885	1174
Sample as proportion of the population in the year of survey (%)	0.003	0.006	0.012
Response rate	56%	-	74.6%
Any adversity	68.2	46.4%	25%
Physical abuse (%)	12.0	14.3	5
Emotional abuse (%)	15.0	17.3	5
Sexual abuse (%)	12.6	6.2	1
	Schilling et al. [51] Hauser et al. [42]	Hughes et al. [43]	present study

**Table 8:** A comparison of prevalence rates (estimates and measured data) of child maltreatment across the globe reported by adults.

Country	Source/ Sample Characteristics	Tools	Prevalence of Child Maltreatment					Prevalence of Household Dysfunctions				
			Physical Abuse	Emotional Abuse	Sexual Abuse (Women, Men)	Emotional Neglect	Physical Neglect	Mental Illness	Substance Abuse	Domestic Violence	Parental Divorce/ Separation	Incarceration
**Worldwide**	Stoltenborgh et al. [52], WHO [53] meta-analysis, 244 publication, 577 estimates	various questionnaires	22.6	36.3	18, 7.6	18.4	16.3	-	-	-	-	-
Africa			22.8	46.7	20.2, 19.3	-	-	-	-	-	-	-
Asia			16.7	41.6	11.3, 4.1	30.1	-	-	-	-	-	-
Australia			14.3	11.3	21.5, 7.5	40.0	-	-	-	-	-	-
North America			24.0	36.5	20.1, 8.0	-	6.5	-	-	-	-	-
South America			54.8	-	13.4, 13.8	14.5	19.2	-	-	-	-	-
Europe			22.9	29.2	13.5, 5.6	18.4	16.3	-	-	-	-	-
EU	WHO [53] meta-analysis, 50 publication, 105 estimates	various questionnaires	22.9	29.2	13.5, 5.6	-	-	-	-	-	-	-
USA (2016)	Taillieu et al. [35], representative sample, N = 34,653	ACE questionnaire five-point ordinal scale	-	4.8	-	6.2	-	-	-	-	-	-
USA (2004)	Dong et al. [21], N = 18,175	ACE questionnaire	26.4	10.2	21	14.8	9.9	20.3	28.8	24.1	13	6
UK (2016)	Hughes, Lowey, Quigg, & Bellis [43]; nationally representative household survey; N = 3885	ACE questionnaire	14.3	17.3	6.2	-	-	12.1	6.5	12.1	22.6	4.1
Germany (2016)	Schilling et al. [51], representative sample N = 2504	German version of the Childhood Trauma Questionnaire (28 items)	2.8	1.6	1.9	6.6	10.8	-	-	-	-	-
Hungary (2017)	Present study representative sample N = 1174	ACE Score Calculator (10-item screening tool)	5	5	1	3	3	6	12	4	13	3

**Table 9:** A comparison of prevalence rates (estimates and measured data) of child maltreatment across the globe reported by children.

Country	Source/Sample Characteristics	Tools	Prevalence of Child Maltreatment					Prevalence of Household Dysfunctions				
			Physical Abuse	Emotional Abuse	Sexual Abuse	Emotional Neglect	Physical Neglect	Mental Illness	Substance Abuse	Domestic Violence	Parental Divorce/Separation	Incarceration
USA (2016)	Turney & Wildeman [54] nationally representative survey N = 95,677 children placed in and adopted from foster care	ACE questionnaire	-	-	-	-	-	8.5	10.5	-	19.9	6.9
Switzerland (1996)	Halpérin et al. [55] nationally representative high-school children	self-constructed questionnaire	20	-	-	-	-	-	-	-	-	-
Romania (2000)	Browne [56] self-report survey children aged 13–14 N = 1295	n.a.	24.0	21.0	9.0	46.0	44.0	-	-	-	-	-
Latvia	Sebre et al. [57], UNICEF [58] multicountry survey; children aged 10–14 Latvia (N = 297) Lithuania (N = 300) Macedonia (N = 302) Moldova (N = 246)	various questionnaires	19.0	29.0	-	-	-	-	-	-	-	-
Lithuania			26.0	33.0	-	-	-	-	-	-	-	-
Macedonia			12.0	13.0	-	-	-	-	-	-	-	-
Moldova			30.0	32.0	-	-	-	-	-	-	-	-

## Appendix A

**Table A1:** The ACE Score Calculator—preambles, item contents and response options.

Item.	Preamble and Content	ACE Category
	During your first 18 years of life:	
1 ^a^	Did a parent or other adult in the household often or very often… Swear at you, insult you, put you down, or humiliate you? or Act in a way that made you afraid that you might be physically hurt?	Emotional abuse
2 ^a^	Did a parent or other adult in the household often or very often… Push, grab, slap, or throw something at you? or Ever hit you so hard that you had marks or were injured?	Physical abuse
3 ^a^	Did an adult person at least 5 years older than you ever… Touch or fondle you or have you touch their body in a sexual way? or Attempt or actually have oral, anal, or vaginal intercourse with you?	Sexual abuse
4 ^a^	Did you often or very often feel that … No one in your family loved you or thought you were important or special? or Your family didn’t look out for each other, feel close to each other, or support each other?	Emotional neglect
5 ^a^	Did you often or very often feel that … You didn’t have enough to eat, had to wear dirty clothes, and had no one to protect you? or Your parents were too drunk or high to take care of you or take you to the doctor if you needed it?	Physical neglect
6 ^a^	Were your parents ever separated or divorced?	Parental separation/divorce
7 ^a^	Was your mother or stepmother: Often or very often pushed, grabbed, slapped, or had something thrown at her? or Sometimes, often, or very often kicked, bitten, hit with a fist, or hit with something hard? or Ever repeatedly hit for at least a few minutes or threatened with a gun or knife?	Household physical violence
8 ^a^	Did you live with anyone who was a problem drinker or alcoholic or who used street drugs?	Household substance abuse
9 ^a^	Was a household member depressed or mentally ill, or did a household member attempt suicide?	Household mental illness
10 ^a^	Did a household member go to prison?	Incarcerated household member

^a^ Dichotomous scales–yes/no.
